# Abdominal Wall Closure of Renal Transplant Recipients: An Undermined Challenge

**Published:** 2010-05-01

**Authors:** A. Halawa

**Affiliations:** *Sheffield Kidney Institute, Northern General Hospital, Sheffield Teaching Hospitals NHS Foundation Trust and University of Sheffield, Sheffield, UK.*

**Keywords:** Renal transplant, muscle closure, Mesh closure, Intraperitonealization, Mesh, Subcutaneous, Transplant compartment syndrome

## Abstract

Tension-free muscle closure is essential in kidney transplantation, both in adult and pediatric patients. Tight muscle closure may lead to renal transplant compartment syndrome either due to compression of the renal parenchyma or due to kinking of the renal vessels. It may also cause kinking of the transplant kidney ureter, wound dehiscence and incisional hernia. Many techniques have been proposed in an attempt to achieve tension-free closure. There is a wrong belief among some surgeons that using prosthetic mesh may increase the incidence of infection complications in these immunosuppressed patients. Also, there is fear that one is not able to monitor the renal graft by ultrasound and perform biopsy in the presence of a mesh. Other alternative techniques to mesh closure include subcutaneous placement and intraperitonealization of the kidney transplant. These techniques however, are valuable when mesh closure is unfavorable or contraindicated as in case of the presence of a potential source of infection like a stoma. Abdominal wall fasciotomy can be adjunctive to various techniques of muscle closure.

## INTRODUCTION

Successful muscle closure following renal transplantation in adults is usually straightforward. However, in some cases, it may present a challenging dilemma to the transplant surgeon. The renal allograft experiences further potential insult after wound closure: ureteral kinking and obstruction, vascular kinking and obstruction or thrombosis, and possibly compartment syndrome secondary to limitation of the retroperitoneal space. These problems are not uncommon in pediatric recipients receiving adult kidneys. It is also encountered in small adult recipients receiving large adult kidneys. Restricted volume of the recipient pelvic cavity and the size discrepancy between the recipient pelvic cavity space and the donor adult kidney may lead to either diffuse renal parenchymal compression or narrowing and/or kinking of the renal veins within a tight compartment [1] causing renal transplant compartment syndrome (RTCS) and subsequent graft thrombosis. Pressure on the graft may be exacerbated by edema secondary to ischemia and/or reperfusion injury in the postoperative period. In either scenario, the end result is a decrease in renal plasma flow and glomerular filtration rate, outflow obstruction with increased intrarenal vascular resistance and edema with subsequent ischemia [2]. There is a strong belief that difficult muscle closure commonly occurs in male peritoneal dialysis patients undergoing retroperitoneal kidney transplantation due to male android pelvis and noncompliance of the peritoneum due to scarring, but this has been proved to be wrong as it also occurs in hemodialysis female patients [3]. Many techniques have been proposed to deal with difficult muscle closure without creating tension and causing compression on the transplanted kidney, both in adult and pediatric transplantation. Abdominal wall fasciotomy can be added as an adjunct to various types of repair to achieve a tension-free closure.

**MESH CLOSURE**

Indeed, many surgeons are reluctant to place synthetic mesh near the renal transplant for fear of infection after ureteroneocystostomy, fistulae, wound dehiscence, interference with biopsy procedure or imaging of the renal graft postoperatively, or inflammatory reaction with resultant perinephric collection. Many types of synthetic mesh closure techniques have been proposed.

Porcine mesh closure

Richards and colleagues (2005) [4] described the use of a porcine collagen graft (Permacol) to facilitate closure of the abdominal wall following intraperitoneal transplantation of an adult cadaveric kidney in a two-year-old male infant weighing 12 kg. The sheet implant was inserted between the recti muscles and sutured to the sheath on either side using continuous PDS. Skin was subsequently closed in the usual fashion. The postoperative course was uncomplicated and the infant was discharged 12 days later. Eighteen months later, the abdominal wound was well healed with no evidence of incisional hernia. Following this successful outcome, this technique has been used in two further cases of pediatric recipients with good results and no evidence of abdominal wall hernia. Pentlow and colleagues (2008) [5] demonstrated a three-year follow-up of five patients aged 5–12 years who received kidneys from adult donors. In four recipients, the kidney was transplanted onto the aorta and vena cava intra-abdominally using a midline incision. In the fifth patient, the kidney was anastomosed onto the iliac vessels. The skin overlying the implant was closed normally. In all cases, primary closure was achieved. One child received a second intra-abdominal transplant as an emergency, which failed later on. The other kidneys are functioning well. One recipient developed a small incisional hernia three years post-transplant. Another developed a skin dehiscence over the implant 23 days post-operatively. The implant was removed and the skin was closed. The other two recipients recovered well. They concluded that porcine dermal collagen implant is a helpful adjunct to abdominal wall closure following organ transplantation in children with donor size discrepancy.

Permacol (Tissue Sciences Laboratories plc, Aldershot, UK) is an acellular sheet of porcine dermal collagen and elastin fibers maintained in their original three-dimensional forms and in which the collagen fibers have been cross-linked using diisocyanate, in order to protect the graft from biodegradation. Porcine dermis is the closest to human dermis in structure and appearance. It is not cytotoxic, hemolytic, pyrogenic or allergenic, does not elicit a foreign body response and is readily colonized by host tissue cells and blood vessels, thus minimizing the risk of infection [6]. It is soft and flexible, yet has high tensile strength and has bilateral smooth surfaces. These properties make it ideal for implantation into sensitive regions. The implant is sold in sheet format in various sizes, allowing it to be cut to shape. The major advantage of porcine dermal collagen implant over conventional synthetic meshes is that it can be used in direct contact with bowel without causing fistulation [7] and causes minimal intraperitoneal adhesions [4]. 

PTFE dual-mesh prosthesis

Maione and colleagues (2006) [8] reported successful management of RTCS in a 42-yr-old renal transplant recipient secondary to extrinsic compression from a large kidney placed extraperitoneally in a small iliac fossa. Prompt re-exploration in the immediate postoperative period resulted in salvage of the graft with restoration of kidney function. The abdominal wall was reconstructed using prosthetic mesh (PTFE), which decreased the compartment pressure within the iliac fossa sufficiently to allow the renal vein patency and the kidney perfusion. The use of a PTFE dual-mesh prosthesis by a tension-free surgical technique allowed avoidance of excessive compression over the kidney inside its new site and to stop RTCS. They suggested that this technique should be the first choice if fascial closure required excessive force in all cases with a considerable size of the graft compared to a quite small pelvis and/or obesity of the recipient. Excessive tension of the aponeurotic edges with a small iliac fossa is a risk for incisional hernia or RTCS. This surgical technique is easy to perform and does not preclude ultrasound evaluation or biopsy of the graft. 

Polypropylene-assisted mesh hood facial closure (MHFC)

**Figure 1:**
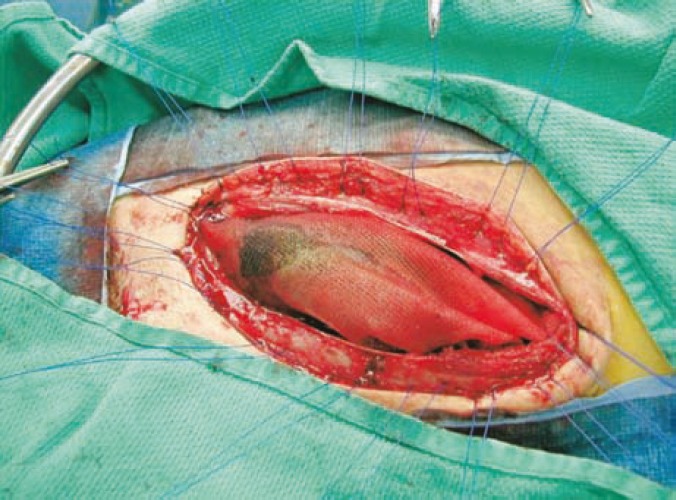
Polypropylene-assisted mesh hood facial closure

Nguan and colleagues (2007) [3] presented their experience in 16 patients undergoing 17 renal transplants who underwent MHFC. The mean follow-up period was nine months. Primary MHFC was performed if fascial closure required excessive force, resulting in a change of graft turgor or color, diminished renal artery pulsation, or change in renal vein turgor. Secondary MHFC was performed when compartment syndrome was suspected postoperatively and confirmed during re-exploration. In most cases, the vessels were straightened by buttressing the hilum using several large folded pieces of gelfoam under the upper and lower poles of the kidney to prevent kinking of the transplanted renal vessels. A large ellipsoid piece of polypropylene mesh was draped loosely and without tension over the graft (Fig. 1). The mesh was attached to the posterior fascial edges using interrupted polypropylene sutures. Skin closure was then completed over a closed suction drain placed in the retroperitoneal space lateral to the kidney. Allograft nephrectomy was performed in one patient without difficulty despite the presence of the previous mesh closure. Ultrasound guided renal biopsy examinations were performed through the mesh closure in five grafts without difficulty. In addition, the MHFC did not provide any hindrance in performing Doppler ultrasound studies on the allograft. Five (31%) patients had prolonged drainage of serous fluid through the wound, resulting in a temporary small area of skin dehiscence in one of the five patients. No wound infections occurred as a result of mesh placement. One patient developed a lymphocele which required drainage. They concluded that MHFC is safe and does not adversely affect the care of the transplant patient, apart from the potential of prolonged wound drainage. They therefore, recommend prolonged closed suction drainage of the subcutaneous space to minimize this complication.

**SUBCUTANEOUS PLACEMENT OF THE KIDNEY TRANSPLANT**

Ball and colleagues (2006) [9] used this technique in three patients diagnosed with RTCS in the early postoperative period. No adverse events were reported with full recovery. All patients had interval hernia repairs with synthetic mesh (mean: eight months). No complications were associated with this type of definitive repair. 

The author also used this technique in two small renal transplant patients receiving large adult cadaveric kidneys. Both kidneys transplanted retroperitoneally into the right iliac fossa. Muscle closure could have caused compression of the graft and subsequent RTCS. No wound-related complications were reported so far with excellent kidney function.

**INTRAPERITONEALIZATION OF THE KIDNEY TRANSPLANT**

Koss and colleagues (2000) [10] reported successful salvage of kidney transplanted into right iliac fossa following tight closure causing RTCS via intraperitoneal graft replacement. Ball, *et al* (2006) [9] also reported eight patients who underwent intraperitonealization of their kidney to treat RTCS. There were no complications associated with intraperitonealization of the renal allograft. Kidney function was recovered with no allograft loss in all cases of RTCS. 

## CONCLUSION

The above techniques are valuable alternatives if tension-free muscle closure could not be achieved. In general, one of these techniques is used at a time. The use of synthetic non-biological mesh (Polypropylene and PTFE) is safe with good results. It is recommended to leave the peritoneum intact when closing with this type of mesh. The other non-mesh techniques and using the biological mesh (Permacol) are very valuable options when placement of a synthetic non-biological mesh is not favorable or contraindicated as in case of presence of potential source of infection like a stoma.
